# Draft genome sequences of eight strains isolated from homemade yogurts and cheeses from Bulgaria

**DOI:** 10.1128/mra.00989-24

**Published:** 2025-01-29

**Authors:** Hanan R. Shehata, Basma Hassane, Andreas Reich, Bojidar Dimitrov Zahariev

**Affiliations:** 1 Purity-IQ Inc., Guelph, Ontario, Canada; 2 Advanced Microbiotics, Stans, Switzerland; Wellesley College Department of Biological Sciences, Wellesley, Massachusetts, USA

**Keywords:** probiotics

## Abstract

Here, we report draft genome sequences of eight strains isolated from naturally processed, homemade dairy foods or human milk in Bulgaria; strains *Lactobacillus gasseri* AM-LG-29, *Lactiplantibacillus plantarum* AM-LP-81, *Lactobacillus helveticus* AM-LH-32, *Lactobacillus delbrueckii* subsp. *bulgaricus* AM-LB-13, *Streptococcus thermophilus* AM-ST-89, *Lactobacillus acidophilus* AM-LA-19, *Bifidobacterium longum* AM-BL-55, and *Lacticaseibacillus rhamnosus* AM-LR-51.

## ANNOUNCEMENT

Here, we present the genome sequences of eight strains isolated from homemade dairy foods in Bulgaria to facilitate research on their probiotic properties since strains of these species have been reported to have probiotic effects. Strains of *L. gasseri* were found to treat irritable bowel syndrome (1) and reduce visceral fat (2). *L. plantarum* strains were reported to relieve ulcerative colitis (3) and improve cognitive functions (4). *L. helveticus* strains were found to have antimicrobial (5) and immunostimulatory effects (6). *L. delbrueckii* subsp. *bulgaricus* strains were reported to have anti-*Helicobacter pylori* activity (7) and to enhance immunity (8–10). *S. thermophilus* was reported to have antimicrobial (11), anti-inflammatory (12), antioxidant, and immunomodulation activities (13). *L. acidophilus* strains proved to exert hypoglycemic and antioxidant activities (14) and antimicrobial activity (15). *B. longum* strains were found to improve chronic constipation (16), alleviate glucose intolerance (17), and improve cognitive functions (18). *L. rhamnosus* strains were reported to have antimicrobial activity (19–21), anti‐inflammatory effects (22), and a role in controlling antibiotic-associated diarrhea (23, 24). Strains AM-LG-29, AM-LP-81, AM-LH-32, AM-LB-13, AM-ST-89, AM-LA-19, and AM-LR-51 were isolated from naturally processed, homemade yogurts and cheeses from ecologically clean preserves, industrial pollution-free and chemically untampered areas of Strandzha/Rhodope/Pirin mountain chains of Bulgaria (Table 1). Strain AM-BL-55 was isolated from first milk of human nursing mothers after giving birth (Table 1).

**TABLE 1:** Sample information, sequencing, and genome assembly information and accession numbers for eight strains isolated from homemade yogurts and cheeses from Bulgaria

Strain	Isolation date	Sample form	Number of reads before quality trimming	Number of reads after quality trimming	Number of bases used for assembly	Coverage	No of contigs	Total draft genome length	Assembly GC (%)	*N*_50_	Number of coding genes	SRA accession no.	Assembly accession no.	The version described in this paper
*L. gasseri* AM-LG-29	May 1999	Lyophilized powder	4,307,196	4,298,171	1,045,637,032	566×	10	1,846,372	35.01	424,129	1,712	SRR29510499	JBEUMA000000000	JBEUMA010000000
*L. plantarum* AM-LP-81	January 1978	Lyophilized powder	4,138,152	4,125,817	992,192,574	310×	20	3,204,092	44.51	365,919	2,944	SRR29510498	JBEUMB000000000	JBEUMB010000000
*L. helveticus* AM-LH-32	February 1981	Lyophilized powder	956,114	945,256	202,189,739	114×	150	1,766,179	36.80	17,643	1,662	SRR29510497	JBEWYM000000000	JBEWYM010000000
*L. delbrueckii* subsp. *bulgaricus* AM-LB-13	September 1969	DNA extract	10,508,930	10,508,804	2,019,654,955	1,136×	49	1,777,087	49.85	74,354	1,684	SRR29510496	JBEWYN000000000	JBEWYN010000000
*S. thermophilus* AM-ST-89	March 1957	DNA extract	18,843,284	18,843,227	4,365,032,937	2,371×	27	1,840,845	38.95	132,639	1,645	SRR29510495	JBEUMC000000000	JBEUMC010000000
*L. acidophilus* AM-LA-19	March 1970	Lyophilized powder	1,420,934	1,406,327	301,389,970	155×	20	1,947,187	34.59	167,370	1,774	SRR29510494	JBEUMD000000000	JBEUMD010000000
*B. longum* AM-BL-55	September 1974	DNA extract	15,016,954	15,016,473	2,521,002,775	1,013×	26	2,488,113	60.24	230,566	2,055	SRR29510493	JBEWYO000000000	JBEWYO010000000
*L. rhamnosus* AM-LR-51	June 1991	Lyophilized powder	4,371,572	4,351,233	976,787,351	332X	30	2,939,766	46.70	149,128	2,635	SRR29510492	JBEUME000000000	JBEUME010000000

All Lactobacilli strains, *S. thermophilus* AM-ST-89, and *B. longum* AM-BL-55 were isolated, respectively, on De Man–Rogosa–Sharpe agar (MRS agar), M-17 agar, and MRS agar supplemented with cysteine, incubated anaerobically at 37°C for 24–48 h (25). All strains were obtained from The National Culture Collection of Switzerland (CCOS) as lyophilized powders or DNA (Table 1). Genomic DNA was extracted from 50 mg of lyophilized powders using NucleoSpin Food kit (740945.50, Macherey Nagel, Germany). DNA was quantified using Qubit 4.0 Fluorometer and submitted to the Advanced Analysis Centre, University of Guelph (Guelph, ON, Canada) for library preparation and sequencing. Samples AM-LG-29, AM-LP-81, and AM-LR-51 were run on Illumina MiSeq using an Illumina Nextera XT kit (Illumina, California, USA) and Illumina MiSeq reagent kit v3 600 cycle (2 × 300 bp reads) (Illumina, California, USA). Samples AM-LH-32, AM-LB-13, AM-ST-89, AM-LA-19, AM-BL-55 were run on Illumina NextSeq 1000 using the Illumina DNA Library Preparation kit and Illumina NextSeq 600 cycle reagent kits (2 × 300 bp reads) (Illumina, California, USA).

CLC Genomics Workbench 24.0.1 (QIAGEN Bioinformatics) was used for sequencing data analysis using default parameters except where otherwise noted. Reads were imported using Illumina High-Throughput Sequencing Import 2.0, quality trimmed using Trim Reads 3.0 (limit = 0.05 base-calling error probability), a maximum of 2 ambiguous nucleotides were allowed, and high-quality reads were assembled using *De Novo* assembly 1.5 using default parameters. Full-length 16S rRNA gene sequence was extracted using ContEst16S (26) and was BLAST searched on GenBank for taxonomic identification (27). The genomes were annotated using NCBI Prokaryotic Genome Annotation Pipeline (PGAP) version 6.7 (28–30) using default parameters. Analysis details are shown in Table 1.

## Data Availability

This Whole Genome Shotgun project has been deposited in DDBJ/ENA/GenBank under the accession numbers in Table 1. The versions described in this paper are the first versions. The raw files were deposited in SRA database under the accession numbers in Table 1.
